# Developing and implementing core competencies in children’s environmental health for students, trainees and healthcare providers: a narrative review

**DOI:** 10.1186/s12909-021-02921-3

**Published:** 2021-09-24

**Authors:** Rose Hannah Goldman, Lauren Zajac, Robert J. Geller, Mark D. Miller

**Affiliations:** 1 Department of Medicine, Cambridge Health Alliance, Cambridge Massachusetts, Cambridge Hospital, Macht Center 427, Cambridge, MA 02139 USA; 2 Department of Medicine, Harvard Medical School, Boston, MA USA; 3 Department of Environmental Health, Harvard T.H. Chan School of Public Health, Boston, MA USA; 4 Department of Environmental Medicine and Public Health, Icahn School of Medicine at Mount Sinai, New York City, New York USA; 5 Department of Pediatrics, Emory University School of Medicine, Atlanta, Georgia; 6 Division of Occupational and Environmental Medicine, Department of Medicine, University of California, California San Francisco, USA

**Keywords:** Environmental health, Pediatric environmental health, Health disparities, Climate change, Medical education, Competencies, Environmental social determinates of health, Environmental justice

## Abstract

Knowledge of the health impacts of environmental exposures (such as pollution disasters, poor air quality, water contamination, climate change) on children’s health has dramatically increased in the past 40 years. The World Health Organization (WHO) estimated that 23% of all deaths worldwide were attributable to the environment, and 26% of deaths in children less than 5 years old could be prevented with removal of environmental risks factors. Yet, little has permeated medical education, leaving pediatric providers ill equipped to address these issues. To address this gap, members from the Pediatric Environmental Health Specialty Units, a United States nationwide network of academically affiliated experts who have created numerous environmental health educational materials and programs, have identified fifteen core environmental health (EH) competencies needed by health care providers to enable them to effectively address environmental health concerns. These competencies can serve as the foundation for the development and implementation of relevant educational programs. The core EH competencies are based upon these foundational elements: 1) Definition of “children’s environmental health” that describes how environmental exposures (positive and negative) in early life influence the health and development in childhood and across the entire human life span 2) Children are not “little adults” and so have unique vulnerabilities to environmental hazards; 3) Environmental health inequities exist, causing some children to have a disproportionate amount of unhealthy exposures and consequently a greater risk of adverse effects; 4) Climate change will translate to numerous adverse health effects that will particularly affect children worldwide. In this article, the authors describe the core environmental health competencies and provide resources, online tools, strategies, and examples targeted to all levels of training and practice to better enable leaders and educators to bring this important content to the forefront.

## Background

Pollution disasters, particularly since the 1980’s, have brought greater attention to the effects of environmental contamination on communities and children. The world’s largest industrial accident, the Union Carbide plant methyl isocyanate gas leak in Bhopal, India in 1984, sent 170,000 people to the hospital, with estimated deaths as high as 8000, including 3000 in those less than 15 years of age, and caused severe health effects in 13,000 small children [1]. In the United States, communities living near hazardous waste sites, such as “Love Canal,” raised concerns about adverse impacts to their children from chronic exposure to polluted environments and toxicants, and formed advocacy groups [2]. An appreciation grew that children may be more vulnerable to toxic environments due to greater opportunities for exposures (given their lower breathing zone, oral exploratory and other behaviors), greater physiological impacts and potential for delayed manifestations [3–6]. A World Health Organization (WHO) 2016 report noted that 23% of all deaths worldwide were attributable to the environment, and 26% of deaths in children less than 5 years old could be prevented with removal of environmental risks factors [5]. Children in industrializing (Low-and Middle-Income Countries, LMIC) suffer from both the traditional environmental health risk factors and diseases (such as indoor air pollution from solid fuels and contaminated water leading to diarrhea, pneumonia and infectious diseases) along with some hazardous exposures more associated with high income countries (HIC) [7, 8]. For example, children in LMIC’s are now exposed to higher levels of outdoor air pollution due to increased combustion of fossil fuels from growing industrial activities and car exhaust accompanying increased car ownership, as well as exposures to metals, e-waste, pesticides, inadequate water availability and quality, and extreme pollution events [7, 9, 10]. Environmental problems for children of HIC’s tend to be linked to chronic, non-communicable diseases that are increasing, such as asthma, neurodevelopmental disorders (dyslexia, attention deficit/hyperactivity disorders, autism), childhood obesity, and cancer (leukemia, brain) [3, 4, 7, 8, 11–14]. For all children, there are concerns regarding the relationship of environmental factors and the COVID-19 pandemic [15], impacts of climate change [16–18], and exposure to manmade ubiquitous long lasting chemicals, such as per- and polyfluoroalkyl substances (PFAS) found in contaminated water and consumer products [19–21].

Children in more low income and minority areas in HIC’s and LIMC’s experience inequalities related to environmental exposures and subsequent adverse outcomes [22]. In the United States, the environmental justice movement raised public awareness about inequities of pollution, racism and effects on communities and children [23, 24]. These forces helped to stimulate the 1997 Executive Order “Protection of Children from Environmental Health Risks and Safety Risks” which charged U.S. federal agencies to consider and address environmental risks to children [25]. In 1998 the Agency for Toxic Substances Disease Registry (Centers for Disease Control and Prevention), an agency tasked with assessing, reducing, and preventing hazardous exposures at Superfund sites, established the Pediatric Environmental Health Specialty Units (PEHSUs). The PEHSUs are a network of experts who provide consultations (telephone and clinic), education, and referral services for reproductive and children’s environmental health (EH) [26]. Further supported by the United States Environmental Protection Agency (USEPA), there are now 10 PEHSU sites across the United States [27, 28]. Comparable entities have been developing in some other countries as well [29].

The concept of children’s environmental health has grown beyond exposures from nearby toxic external environments to encompasses those aspects of children’s health, including quality of life, that are determined by interactions with physical, chemical, biological and social factors in the environment [3]. The field focuses on environmental exposures that span the period from parents’ pre-conception through pregnancy, infancy, childhood, and adolescence. It includes inhaled air, as well as ingested water and food, housing conditions, consumer products, e-waste and other materials encountered in one’s built or social environments as well as the effects of climate change.

It is imperative that clinicians taking care of children and pregnant women, whether in HIC’s or LMIC’s, improve their environmental health literacy, access and utilize existing resources that address factors in their relevant environments, be able to take an environmental history, give anticipatory guidance, and in some cases, act as a resource for their communities [3, 7, 30]. The American Academy of Pediatrics (AAP) developed the Council On Environmental Health to address the effects of radiation, chemicals and environment in general, and has created important publications [3]. WHO has created training modules related to children’s exposures to various agents, such as heavy metals, radiation, and noise, and ones that address broader environmental health topics such as developmental and environmental origins of adult disease and the pediatric environmental history [31]. The US EPA and National Institute of Environmental Health Sciences (NIEHS)-sponsored Children’s Environmental Health Research Centers have expanded the scientific evidence base of children’s unique vulnerability to environmental toxicants [32].

Despite these efforts, deficiencies have been reported and still exist in the training of physicians and other health care providers regarding environmental health factors, starting at the medical school curriculum level [33–37], and extending to those in practice. For example, pediatricians surveyed in the U.S. state of Georgia in 2002 reported that they strongly believed in the importance of environmental exposures in children’s health, yet less than one in five received training in taking the environmental health history [38]. Similarly, pediatric oncology providers at a variety of U.S. sites rarely take a history about environmental exposures and are uncomfortable answering questions about them from their patient’s families [39]. In a study of 72 Egyptian pediatricians attending two education sessions 1 month apart, over half reported some awareness of pediatric health topics, but very low appreciation of the value of the environmental health history in practice [40]. A post education intervention survey revealed a higher proportion were including questions about environmental toxic exposures and parents’ occupation in the medical history and revealed the need for further awareness and attention to pediatric environmental health issues in practice [40].

Our paper addresses this education gap by first identifying core competencies needed by health care providers to enable them to effectively address EH concerns and issues (Table 1). These EH competencies can then serve as the foundation for development and implementation of relevant educational programs, targeted to all levels of training and practice, and to location priorities. The EH competencies in the areas of foundational knowledge, patient care and community health are reflective of established core competencies in medical education that address population health, including social and environmental determinants of health, community health, and health disparities [41]. We also provide examples and resources to assist in fulfilling these competencies that can be applied at various levels of training and practice.

**Table 1:** Core Competencies in Children’s Environmental Health

Core Competency Area	Core Competency Learning Objectives	Methods of Assessment
**1. Medical Knowledge**	a. Explain how fetal and childhood epigenetics, physiology, organ maturation, and behaviors at different stages of development increase the impact of environmental exposures b. Identify potentially relevant EH resources for screening, diagnosis, risk communication, and treatment c. Describe prenatal, perinatal and pediatric conditions that are linked to environmental factors d. Describe prenatal, perinatal and pediatric exposures that may lead to disorders in birth outcomes, childhood, adolescence, and/or adulthood e. Describe aspects of the environment that nurture healthy growth and development in the fetus, child, adolescent, and young adult	Exam questions in pre-clinical years Case studies in problem-based learning Standardized patient encounters Oral questions on patient rounds or educational sessions Pre and post tests for Continuing Education activities Participation in EH sessions at academic conferences
**2. Patient Care**	a. Apply environmental health screening questions as developmentally appropriate in preventive care b. Utilize additional necessary relevant questioning or assessment, if a potential hazard is identified c. Incorporate questions about potentially relevant environmental causes/factors in the assessment of the sick child d. Apply appropriate environmental and biological sampling and measurement both for screening and diagnostic testing e. Create and implement appropriate environmental health risk counseling and hazard reductions f. Demonstrate skills in providing environmental health anticipatory guidance	EH case studies in problem-based learning Standardized patient encounters with EH-related concerns EH content in morning report and other educational sessions Quality improvement projects, Maintenance of Certification activities focused on EH topics Pre and post tests for EH Continuing Education activities
**3. Systems-based Practice (Community Health)**	a. Describe sentinel events and strategies for their public health reporting b. Identify partners and resources for addressing environmental health risks, and for counseling and management at the individual and community level. c. Demonstrate skills in EH risk communication d. Identify legal, regulatory, and non-regulatory approaches to addressing EH issues.	EH and public health elective participation for students and residents Scholarly project/research opportunities Participation in professional organization or institutional committee/working group addressing social or environmental determinants of health Refer patients to community-based resources

## Foundation of the core competencies in children’s environmental health

A committee of pediatric environmental health experts from the PEHSU network developed core competencies in children’s environmental health, based upon these foundational elements:

### Definition of “children’s environmental health”

Children’s environmental health has been defined as “the academic discipline that studies how environmental exposure in early life--chemical, nutritional, and social--influence health and development in childhood and across the entire human life span” [12]. Environmental health in clinical practice involves diagnosis, treatment and prevention of illnesses associated with harmful exposures in the environment, but also encompasses fostering the healthy aspects of an environment that are important for a child’s development, such as opportunities for exercise and the safe exploration of surroundings and nature [3, 42]. There is also growing evidence that social stressors can act synergistically with chemical exposures to harm children’s health and development [43, 44]. Environmental exposures are relevant not only to general pediatrics but also to subspecialties such as pediatric nephrology, cardiology, neurology/developmental, pediatrics pulmonary and oncology [45–48].

### Children are not “little adults” [3, 4]

Children have unique “vulnerabilities” to environmental hazards, that vary with their developmental stage, behavior, and types of activities, and involve such factors as routes of exposure, metabolism, oxygen consumption, respiratory rates and target organ susceptibility and developmental “windows of vulnerability” [3, 4, 12].

### Environmental justice and health inequities

Some children have a disproportionate share of unhealthy exposures in their environment, and consequently a greater risk of adverse effects [22, 23]. Many of these children lack access to healthy homes and physical environments (some resulting from historical discriminatory practices), as well as enriched and supportive social environments that would contribute to healthy development. Environmental justice and health inequalities is a global problem [49]. These children, who are confronted by the cumulative impacts of unhealthy exposures and limited services, face barriers in overcoming these exposures, and consequently suffer negative health outcomes [50].

### Climate change and children’s health

Climate change is bringing about different changes in different regions of the globe that will translate to numerous adverse health effects as the results of disasters, increased heat stress, worse storms, floods, draught, decreased air quality, food and water shortages, and psychological stress, and children will be particularly vulnerable to these threats [16, 17, 51, 52]. Pediatricians can play an important role as protectors of the health and safety of children, and as advocates for changes that address both the mitigation and adaptation to climate change [17, 53, 54] . Climate change has been highlighted as an important topic for medical school and residency curricula [55–57].

In consideration of these factors, Table 1 presents proposed competencies in the categories of medical knowledge, patient care, and systems-based practice (community health).

## Environmental health resources and experiences that fulfill the core competencies

In the last decade, more educational materials, clinical experiences, and online tools related to pediatric environmental health have been developed to fulfill EH competencies. These resources have contributed to the education of medical students, nurses, health professionals, trainees and providers, and serve as models for the development of future experiences and materials (Table 2: Resources). The following is a brief description of some of these pediatric EH resources and initiatives.

**Table 2:** Pediatric Environmental Health Resources

**Clinical Consultations for Physicians**	
**Pediatric Environmental Health Specialty Unit (PEHSU)** www.pehsu.net Experts at your regional PEHSU can provide consultations on the prevention, diagnosis, and management of environmentally-related illness.	
**Evidence-Based EH Information to Use in Clinical Practice**	
**Pediatric Environmental Health Toolkit** peht.ucsf.edu Online reference tool (mobile-device friendly) for evidence-based information on environmental hazards, prevention strategies, and patient resources. Endorsed by AAP, Physicians for Social Responsibility, PEHSU.	
**Prescriptions for Prevention** http://nyscheck.org/rx Evidence-based concise information for families, available in English and Spanish. The front of each prescription explains simple steps for reducing the exposure, and the back lists key resources that families can use to help address the concern.	
**National Environmental Education Foundation (NEEF)** www.neefusa.org/health-wellness EH history forms (general and for asthmatics) in English and Spanish.	
**AAP: Pediatric Environmental Health (“The Green Book”)** http://ebooks.aappublications.org/content/pediatric-environmental-health-4th-ed	
**AAP: EH Policy Statements and Technical Reports** pediatrics.aappublications.org/collection/council-environmental-health	
**Environmental Risks to Children: Prioritizing Health Messages in Pediatric Practice** Galvez and Balk. *Pediatrics in Review*. 2017Jun1;38(6):263–79.	
**EH Data Resources**	
**CDC National Report on Human Exposure to Environmental Chemicals** https://www.cdc.gov/exposurereport Summary biomonitoring data on 352 environmental chemicals in a nationally representative sample of the US population (1999 through 2016; using NHANES study population)	
**CDC National Environmental Public Health Tracking Network** https://www.cdc.gov/nceh/tracking/ Standardized health, environmental, and hazard data from multiple information systems with easy-to-explore data portals that allow for customized maps, tables, and charts of local, state, and national data.	
**WHO. Ambient air pollution attributable deaths** https://www.who.int/data/gho/data/indicators/indicator-details/GHO/ambient-air-pollution-attributable-deaths Interactive map with data	
**AirNow** https://www.airnow.gov/ Interactive maps that provide current air quality data where you live, U.S. as well as internationally. Also Fire and Smoke Maps	
**IPCC Working Group 1, Sixth Assessment Report Interactive Atlas** https://interactive-atlas.ipcc.ch/ A flexible spatial and temporal analyses to see the observed and projected climate change regional information	
**Educational Modules for Trainees and Practitioners**	
**Pediatric Environmental Health Specialty Unit (PEHSU)** www.pehsu.net PEHSU website has a national “classroom” with online course, webinars (offering Continuing Education Credit) and factsheets, could be applied to trainees and providers. Some fact sheets translated into Spanish.	
**Pediatric Environmental Health Interactive Curriculum** https://wspehsu.ucsf.edu/main-resources/for-clinical-professionals/training/pediatric-environmental-health-interactive-curriculum/ This is an easy to administer environmental health curriculum, which meets ACGME requirements, and aims to teach pediatric residents’ proficiency in environmental health and establish a foundation of knowledge upon which they will expand during their careers.	
**A Story of Health** https://wspehsu.ucsf.edu/main-resources/for-clinical-professionals/training/a-story-of-health-a-multi-media-ebook/ Multimedia eBook that explores how environments interact with a person’s genes to influence health across the lifespan, told through the lives of fictional characters and their families.	
**Children’s Environmental Health Network (CEHN)** cehn.org/resources/for-healthcare-professionals/ This multidisciplinary advocacy organization provides Power point modules and user guides to incorporate pediatric EH into training. It also has training modules for child care professionals.	
**WHO Training Modules and instructions for health care providers** **https://www.who.int/teams/environment-climate-change-and-health/settings-populations/children/capacity-building/training-modules** Power point type slide decks, with notes and instructions on numerous topics related to children environmental health which can be used for self-directed learning and/or adapted for teaching at any level of student or professional. Also provided is an example of post-workshop knowledge evaluation as a download	
**The Medical Society Consortium on Climate and Health on Climate & Health** https://medsocietiesforclimatehealth.org/educate/ Comprehensive teaching materials and resources relevant to climate change, for teaching and learning at all levels: health educators (for health professional student-slide shows, links to courses, nine short slide decks) other teaching resources targeted to health professionals (recordings and slide decks), patients, and community, as well as Webinars for CME	

Health care providers can access online tools, even during a patient encounter, such as the Pediatric Environmental Health Toolkit (PEHT), endorsed by American Academy of Pediatrics (AAP) [58]. This resource provides succinct evidence-based information which includes provider and patient educational materials tailored to developmental stages. One hundred pediatric providers and trainees who were trained to use the PEHT in different primary care settings, reported substantial improvements in provider knowledge and confidence with counseling about EH issues. After 6 months of use, most participants responded that they “intended to continue to use the materials,” (96%) and advise patients on environmental health issues more often (86%). The original PEHT has been converted to a web-app for wider availability. Of the more than 13,000 users over the past 2 years (August 2019–2021), greater than 500 were from 60 countries outside of North America, indicating a broad international interest in these kinds of products.

In 2019 AAP released the 4th edition of the “Green Book” *Pediatric Environmental Health,* in both paper and electronic versions, which provides practical information for both clinical care, patient education, and teaching [3]. Galvez and Balk provide an outline of select environmental health priority areas for clinicians, along with key environmental health questions and messaging that can be incorporated into appointments [30].

The Pediatric Environmental Health Specialty Unit (PEHSU) web site offers free access to numerous resources (fact sheets, webinars, electronic eLearning modules), some translated into other languages, as well as contact information to regional experts [26]. As of August, 2021, the PEHSU National Classroom, launched in 2015, has provided 99 webinars and 26 eLearning modules, covering many of the core competencies. Over 21,000 participants (from the U.S, Canada, Israel, Australia, India and Spain), including nurses, residents, practitioners from pediatrics, family medicine, toxicology, emergency medicine, obstetrics/gynecology, medical and nursing students, paramedics, pharmacists and others have completed post-test assessments, which consisted of at least 5 questions (multiple choice and true/false) based on the learning objectives for each activity, with 80% receiving a passing score of 80% correct. For 12 interactive modules, 994 participants (representing various levels of physicians and students and non-clinical professionals) completed pre-tests and post-tests questionnaires on at least one module (5–15 multiple choice or true /false questions) [59]. Post-test scores were significantly higher than pre-test scores, demonstrating an improvement of 30.74%. There was a small response to a 6-month post-test survey, and so this assessment was not possible to determine the actual clinical impact of the eLearning modules on actual clinical practice [59].

Medical institutions such as University of California San Francisco (UCSF), Harvard Medical School (HMS), and the Icahn School of Medicine at Mount Sinai (ISMMS) offer EH electives that provide experience with the prevention, diagnosis, and management of EH concerns in the clinical and community settings. The Western States PEHSU (WS-PEHSU) developed an Interactive Pediatric Environmental Health Curriculum, an easy to administer environmental health curriculum, which meets ACGME requirements, is adaptable to multiple educational sites, and does not require expertise in environmental health to administer (a comprehensive faculty guide is provided) [60]. It includes three modules: a neighborhood visit to explore community environmental factors, a home visit to explore the intersection of housing and health, and a critical evaluation of information in a mainstream publication. The New England PEHSU developed a case-based interactive lecture on pediatric environmental health that was given at Simmons School of Nursing, and then developed into an online module for their students. The ISMMS elective has adapted to offer a virtual elective experience during the COVID-19 pandemic, including telephone screening of high-risk patients for environmental and social determinants of health.

Narrative approaches and storytelling are powerful health promotion tools that can increase understanding of determinants of health, translate complex science, and improve care. *A Story of Health* (SOH), a multimedia eBook, harnesses the power of storytelling to improve environmental health literacy for health professionals, health educators and advocates [61]. It addresses multiple environmental contributors to health and illness and their interactions across the lifespan in chapters on asthma, developmental disabilities, childhood leukemia, infertility/reproductive health, and cognitive decline. By grounding the science of health in stories of fictional people, their families, and communities, readers can explore risk factors and prevention strategies. Chapter stories address environmental justice and communities of color to encourage and improve “culturally responsive care.” The eBook is intended to foster the use of anticipatory guidance around environmental exposures in clinical and community practice. Over 16,000 health professionals have registered through the CDC for free CE credits using the Story of Health module. In the feedback survey, more than 85% of users have reported they will incorporate the information into their practice and provide interventions [61]. SOH has been adapted for use in clinical education of doctors, nurses and public health professionals.

## Strategies to build competence

It would be unrealistic to expect every graduating medical/health professional student and practitioner to be an expert in environmental medicine. However, by introducing key EH topics in a more substantial way during pre-clinical and clinical years for trainees, and through continuing education for practitioners, healthcare providers could become better prepared to address parent and patient concerns, access EH resources, address exposures, and provide anticipatory guidance on EH. Given the climate crisis, and its multiple effects particularly on children, it is imperative for institutions training health professionals to increase their educational initiatives related to climate change [55, 57, 62] The following are some examples of existing strategies for integrating EH that can increase foundational knowledge, enhance clinical skills, and address community health concerns.

1. Preclinical foundational coursework: Integrate EH topics into relevant existing curriculum sessions and associated assessment (e.g. written examinations, problem-based learning exercises). Some examples include a discussion of environmental triggers and air pollution’s impact on lung development in pulmonology sessions on asthma, discussion of endocrine disrupting chemicals in relevant endocrinology course, and discussion of climate change-related expanding range of vector-borne diseases in infectious disease courses.
2. Clinical clerkships and residency training: Medical schools and residencies have begun to incorporate social determinants of health and advocacy training in clerkships and residencies across specialties [63–65]. EH (including environmental justice) is an important component to include among the social determinants and can be a focus for advocacy training and case studies. For clerkships and residencies, it is important to teach fundamentals, such as taking an environmental history and a targeted environmental history for key conditions such as asthma (environmental triggers) and lead poisoning. Standardized patient (SP) cases and assessments could be created that include contributing environmental factor that could be relevant to that location. In one example, a SP case used in a medical student clerkship involved a person with asthma exacerbation triggered by wildfire, which stimulated increased awareness of health impacts of climate change on individual patients, and connections to public health actions such as mitigation and adaptation [66].

For residency, EH topics could be included in numerous ways, incorporating case studies into Morning Report, ambulatory wrap up rounds and other resident educational sessions. Philipsborn et al. [57] provide a comprehensive framework for how to include relevant climate change topics into residency education. Such a framework could be applied to other EH topics. Clinical preceptors can encourage the inclusion of environmental histories as routine part of patient care, both as part of diagnostic reasoning as well as for preventive care (example: screening for radon).

3. Curriculum enhancement for medical (and other health profession) students. Medical Centers with established EH expertise and faculty, can create and offer EH electives for medical students or residents (example curricula are available). The centers can also provide opportunities for students to get involved in EH research or service projects, participate in rotations with clinical or environmental public health faculty. For Centers without an EH program, encourage trainees to participate in away electives, rotations or projects at established programs as offered, for example, through the PEHSU network. Trainees and providers can join committees or working groups based in a professional organization (e.g., AAP Council on Environmental Health) or through their local institutions (e.g., Social Determinants of Health Committee).
4. Postgraduate training opportunities: A Pediatric Environmental Health Fellowship is available at several institutions [26, 67]. Additional training opportunities exist for non-physician allied health professionals, such as the Association of Schools & Programs of Public Health Environmental Health Fellowship Program (ASPPH/USEPA [68] and the Environmental Health Nurse Fellowship [69].
5. Continuing Medical Education (CME) and Maintenance of Certification (MOC) for practicing clinicians, and other Continuing Education requirements: Practitioners in various aspects of patient care are usually required to perform continuing education. EH topics could be incorporated into local “Grand Rounds”, departmental lectures, professional conferences, and online continuing education learning modules. For example, providers can access and participate in existing CME events and modules as provided on the PEHSU national classroom [26]. In the U.S., pediatric organizations can offer MOC Part 2 or Part 4 credits that focus on EH issues. The AAP offers EH-related Project ECHO (Extension for Community Health Care Outcomes) Series.
6. Clinical tools available to help integrate EH into routine patient care: Providers can utilize existing tools to enhance capacity to address EH in clinical care (see Table 2). As clinics are beginning to incorporate social determinants of health into the electronic medical record (EMR) [70, 71], efforts could be made to include relevant EH history questions and anticipatory guidance.
7. Partnerships: It is also possible to leverage relationships with key partner organizations to include EH topics into existing meetings and conferences (e.g., AAP Chapters (USA), State Medical Societies, Public Health Conferences, Nursing Events and other professional organizations in relevant geographic locations).

## Potential barriers to implementing the competencies

Challenges for insertion and implementation of these competencies and curricula into medical school and health care curricula include the already pre-existing compression of medical school curricula and lack of appreciation of this topic as an important component for medical education, as well as the shortage of knowledgeable champions who have access and input into the curricula. Although there is limited funding for development of new materials, there are already numerous resources that could be deployed, with sufficient recognition of the need of this important education.

## Future directions

A major goal is to utilize these core pediatric competencies and framework to stimulate and guide relevant learning initiatives in a variety of geographical locations worldwide. Increasingly the PEHSU members and staff have been involved in consultations and educational programs with colleagues in many countries, including Korea, Japan, Israel, Spain, Chile, Argentina, Vietnam, Mexico and others [29]. To support these efforts, the PEHSU network has been creating public educational materials in multiple languages. For example, the Safer Disinfectants fact sheet has been translated in Korean, Portuguese (Brazilian), Russian, Somali, Spanish Vietnamese and more. Resources that are in English could be easily adapted to other specific languages, cultural and needs of other countries.

The USEPA and PEHSU program have worked in other countries to promote an adoption or adaptation of a PEHSU model that comprises a network of experts and staff who can offer medical education, outreach, and consultative services to communities and other health professionals. There have been presentations regarding environmental health and the PEHSU model at international meetings sponsored by the International Pediatric Associations (IPA) and International Society for Environmental Epidemiology (ISEE).

More can be done to identify “champions” of children’s environmental health in various locations and institutions, who can then inspire and train others [72].

## Conclusion

It is critical that practitioners who care for children have the knowledge and experience to address pediatric environmental health factors. Our list of competencies, coupled with access to available EH resources, provide a structure for organizing education and resources to meet the needs of all levels (medical students, residents, practitioners, allied health care providers). Many of the key pediatric environmental health topics can be woven into existing curriculum and structures for medical students and residency training, and into continuing education courses for practitioners. Leaders and educators at all levels need to take a more active role in bringing this content to the forefront worldwide.

## Data Availability

Not applicable since no references made to data sets.

## Abbreviations

AAP: American Academy of Pediatrics
PEHT: Environmental Health Toolkit
PEHSU: Pediatric Environmental Health Specialty Units
PFAS: Polyfluoroalkyl substances
EH: Environmental health
